# Association of Flavor Perception With Blue vs Purple Cigar Packaging Among US Adults

**DOI:** 10.1001/jamanetworkopen.2022.54003

**Published:** 2023-02-01

**Authors:** Cristine D. Delnevo, Ollie Ganz

**Affiliations:** Rutgers Center for Tobacco Studies, Rutgers Biomedical and Health Sciences, New Brunswick, New Jersey; Department of Health Behavior, Society and Policy, Rutgers School of Public Health, Piscataway, New Jersey; Rutgers Center for Tobacco Studies, Rutgers Biomedical and Health Sciences, New Brunswick, New Jersey; Department of Health Behavior, Society and Policy, Rutgers School of Public Health, Piscataway, New Jersey

## Introduction

A leading US cigar brand Black & Mild recently emailed customers to promote its newest blend, Royale, in purple packaging. Previously, the company sold the same cigar in a blue pack (promoted on its website in 2011–2016). This shift is notable since pack color influences consumer perceptions, including of flavor,^1–3^ and certain colors are associated with specific flavors (eg, green with menthol).^2^ We hypothesized that consumer flavor perceptions differ for blue vs purple cigar packaging.

## Methods

The Rutgers University Institutional Review Board deemed this online survey study exempt because of minimal participant risk. Participants provided consent before survey completion. The study followed the AAPOR reporting guideline.

We examined the association of cigar pack color with consumer flavor perceptions using data from 1 wave of the Rutgers Omnibus Study (a quarterly Amazon Mechanical Turk [mTurk] survey of US adults aged 18–45 years) collected in August 2022. Respondents were randomized to view a cigar with blue or purple packaging and asked if the cigar was flavored (yes or no) and, if yes, what flavor. We used multivariable logistic regression models to examine the association of condition (pack color) with cigar use, self-reported demographic characteristics (age, sex, and race and ethnicity), and flavor perceptions. Statistical significance was defined as *P* < .05 (2-tailed) and analyses were conducted in October 2022 using Stata/MP, version 17 (StataCorp).

## Results

There were 2541 survey respondents, including 1546 female (60.8%) and 979 male (38.5%) individuals (Table 1). The mean (SD) respondent age was 33.1 (6.6) years; 278 (10.9%) self-identified as Hispanic, 289 (11.4%) as non-Hispanic Black, 1722 (67.8%) as non-Hispanic White, and 235 (9.2%) as other non-Hispanic race. There were 1035 ever cigar users (40.7%) and 457 current users (18.0%). More participants randomized to purple vs blue believed that the product was flavored (69.2% vs 50.8%; Table 2). Independent of condition, previous cigar smoking and female sex were associated with believing that the cigar blend was flavored. Significant differences were observed for perceived flavors by color, with blue evoking vanilla (adjusted odds ratio, 2.87; 95% CI, 2.09–3.95), blueberry (7.33; 4.16–12.92), or mint/menthol (2.61; 1.59–4.29) and purple evoking grape (11.37; 8.21–15.73), wine (8.32; 4.22–16.39), or berry/cherry (5.72; 3.74–8.72). Flavor perceptions for blueberry, grape, and wine were independently associated with cigar smoking.

## Discussion

Although previous studies have investigated the association of cigar pack color with consumer perception,^1–3^ our survey study is the first, to our knowledge, to report its association with specific flavor perceptions. The results suggested that purple communicated the product was flavored more so than blue. While this perception was universal regardless of respondent cigar smoking status, it was more common among cigar smokers and likely reflects their familiarity with the flavored cigar marketplace.

Cigar smokers tended to indicate specific flavors that reflected existing color and flavor combinations (eg, blue/vanilla). This is known as sensation transference, a marketing strategy used by tobacco companies.^4^ Individuals who use the brand we studied reported higher odds of perceiving purple as wine (a popular flavor), whereas never cigar users and users of other brands rarely suggested wine. Grape, one the most popular cigar flavors, was the most endorsed perceived flavor for purple packaging regardless of cigar smoking status.

The use of mTurk is a study limitation, as mTurk is a nonprobability online survey and therefore may underestimate or overestimate prevalence. Indeed, current cigar use prevalence was higher in our sample compared with national estimates,^5^ although definitions of “current use” differ. However, mTurk samples yield generalizable findings for split-sample experiments such as ours.^6^ In addition, we focused on a single cigar brand; thus, our findings may not generalize to other brands.

Our findings are timely, given that the US Food and Drug Administration (FDA) plans to ban flavored cigars. Pack color will likely become increasingly important for tobacco companies to communicate product attributes to consumers. Our study suggests that color can convey distinct messages to different audiences, including product users and nonusers, and to users of specific brands. Further, given the association of pack color with perceived flavor, the FDA should consider tobacco products with pack-color changes as new products that are subjected to rigorous product review.^2^

## Supplementary Material

data sharing statement

## Figures and Tables

**Table 1.** Sample Characteristics Overall and by Cigar Experiences

Characteristic	No. of respondents (%)			
	Overall (N = 2541)	Ever use of cigars (n = 1035)	Current use of cigars (n = 457)	Current B&M user (n = 126)
Age, y				
18–25	366 (14.4)	93 (9.0)	64 (14.0)	10 (7.9)
26–35	1177 (46.3)	483 (46.7)	228 (49.9)	75 (59.5)
36–45	998 (39.2)	459 (44.3)	165 (36.1)	41 (32.5)
Missing	0	0	0	0
Sex				
Male	38.5 (979)	401 (38.7)	248 (54.3)	70 (55.6)
Female	60.8 (1546)	632 (61.1)	202 (44.2)	55 (43.6)
Missing	0.6 (16)	2 (0.2)	7 (1.5)	1 (0.8)
Race and ethnicity				
Hispanic	278 (10.9)	112 (10.8)	60 (13.1)	18 (14.3)
Non-Hispanic Black	289 (11.4)	90 (8.7)	75 (16.4)	29 (23.0)
Non-Hispanic White	1722 (67.8)	748 (72.3)	278 (60.8)	70 (55.6)
Other non-Hispanic race^a^	235 (9.2)	83 (8.0)	37 (8.1)	8 (6.3)
Missing	17 (0.7)	2 (0.2)	7 (1.5)	1 (0.8)
Cigar use^b^				
Current	457 (18.0)	NA	NA	NA
Ever	1035 (40.7)	NA	NA	NA
Never	1049 (41.3)	NA	NA	NA
Missing	0	NA	NA	NA
B&M smoker^c^				
Yes	126 (5.0)	NA	NA	NA
No	2415 (95.0)	NA	NA	NA
Missing	0	NA	NA	NA

Abbreviations: B&M, Black & Mild; NA, not applicable.

a Other non-Hispanic race and ethnicity included American Indian or Alaska Native, Asian, Native Hawaiian or Pacific Islander, or other not specified here.

b Categories are mutually exclusive and were defined as follows: never use of any cigar product (ie, traditional or premium, filtered, or cigarillo), ever but not current use of any cigar product, or current use of any cigar product (use every day, some days, or rarely).

c Defined as those who reported B&M as their regular or last brand for any cigar product (ie, traditional or premium, filtered, or cigarillo); all B&M smokers were current smokers.

**Table 2.** Association of Pack Color With Cigar Use Behaviors, Demographic Characteristics, and Flavor Perceptions

Characteristic (No. of respondents) (N = 2541)	Believes product is flavored		Perceived flavor											
			Vanilla		Blueberry		Mint/menthol		Grape		Wine		Berry/cherry	
	No. (%)	AOR (95% CI)^a^	No. (%)	AOR (95% CI)	No. (%)	AOR (95% CI)	No. (%)	AOR (95% CI)	No. (%)	AOR (95% CI)	No. (%)	AOR (95% CI)	No. (%)	AOR (95% CI)
Condition														
Blue (n = 1319)	670 (50.8)	1 [Reference]	155 (11.8)	2.87 (2.09–3.95)	102 (7.7)	7.33 (4.16–12.92)	60 (4.6)	2.61 (1.59–4.29)	46 (3.5)	1 [Reference]	10 (0.8)	1 [Reference]	29 (2.2)	1 [Reference]
Purple (n = 1222)	846 (69.2)	2.22 (1.88–2.62)	5 (4.5)	1 [Reference]	14 (1.2)	1 [Reference]	22 (1.8)	1 [Reference]	346 (28.3)	11.37 (8.21–15.73)	68 (5.6)	8.32 (4.22–16.39)	131 (10.7)	5.72 (3.74–8.72)
Cigar use^b^														
Never (n = 1049)	535 (51.0)	1 [Reference]	82 (7.8)	1 [Reference]	30 (2.9)	1 [Reference]	35 (3.3)	1 [Reference]	110 (10.5)	1 [Reference]	15 (1.4)	1 [Reference]	56 (5.3)	1 [Reference]
Ever (n = 1035)	667 (64.4)	1.74 (1.45–2.09)	89 (8.6)	1.15 (0.84–1.59)	51 (4.9)	1.95 (1.21–3.12)	29 (2.8)	0.79 (0.47–1.32)	211 (20.4)	2.31 (1.77–3.03)	35 (3.4)	2.32 (1.24–4.32)	72 (7.0)	1.27 (0.87–1.84)
Current (n = 457)	314 (68.7)	1.86 (1.42–2.43)	39 (8.5)	1.10 (0.69–1.77)	35 (7.7)	2.77 (1.53–5.00)	18 (3.9)	0.98 (0.51–1.91)	71 (15.5)	1.83 (1.25–2.66)	28 (6.1)	2.77 (1.28–6.01)	32 (7.0)	1.30 (0.77–2.20)
B&M smoker^c^														
Yes (n = 126)	93 (73.8)	1.43 (0.90–2.28)	13 (10.3)	1.33 (0.65–2.71)	14 (11.1)	1.52 (0.71–3.22)	5 (4.0)	0.86 (0.30–2.51)	14 (11.1)	0.63 (0.32–1.22)	15 (11.9)	3.68 (1.63–8.29)	8 (6.4)	1.10 (0.47–2.60)
No (n = 2415)	1423 (58.9)	1 [Reference]	197 (8.2)	1 [Reference]	102 (4.2)	1 [Reference]	77 3.2	1 [Reference]	378 (15.7)	1 [Reference]	63 (2.6)	1 [Reference]	152 (6.3)	1 [Reference]
Sex^d^														
Male (n = 979)	619 (63.2)	1 [Reference]	73 (7.5)	1 [Reference]	34 (3.5)	1 [Reference]	46 (4.7)	1 [Reference]	154 (15.7)	1 [Reference]	34 (3.5)	1 [Reference]	58 (5.9)	1 [Reference]
Female (n = 1546)	890 (57.6)	0.84 (0.71–1.00)	137 (8.9)	1.28 (0.94–1.74)	81 (5.2)	1.83 (1.19–2.81)	36 (2.3)	0.47 (0.30–0.74)	237 (15.3)	0.99 (0.78–1.26)	44 (2.9)	0.93 (0.57–1.51)	100 (6.5)	1.13 (0.80–1.60)
Race and ethnicity^e^														
Hispanic (n = 278)	173 (62.2)	1.08 (0.83–1.42)	25 (9.0)	1.06 (0.68–1.67)	14 (5.0)	1.09 (0.60–1.99)	11 (4.0)	1.53 (0.78–3.04)	54 (19.4)	1.38 (0.96–1.97)	6 (2.2)	0.61 (0.25–1.48)	16 (5.8)	0.83 (0.48–1.44)
Non-Hispanic Black (n = 289)	178 (61.6)	1.14 (0.88–1.50)	19 (6.6)	0.69 (0.41–1.14)	21 (7.3)	1.39 (0.82–2.35)	15 (5.2)	2.22 (1.20–4.11)	41 (14.2)	1.04 (0.70–1.53)	12 (4.2)	1.31 (0.66–2.60)	13 (4.5)	0.67 (0.37–1.22)
Non-Hispanic White (n = 1722)	1020 (59.2)	1 [Reference]	147 (8.5)	1 [Reference]	76 (4.4)	1 [Reference]	46 (2.7)	1 [Reference]	268 (15.6)	1 [Reference]	52 (3.0)	1 [Reference]	118 (6.9)	1 [Reference]
Other non-Hispanic race (n = 235)^f^	137 (58.3)	0.99 (0.74–1.32)	19 (8.1)	0.97 (0.58–1.60)	4 (1.7)	0.40 (0.14–1.13)	10 (4.3)	1.58 (0.78–3.22)	27 (11.5)	0.70 (0.44–1.09)	8 (3.4)	1.15 (0.52–2.52)	11 (4.7)	0.68 (0.36–1.29)

Abbreviations: AOR, adjusted odds ratio; B&M, Black & Mild.

a Adjusted analyses controlled for age and all variables presented here.

b Defined as follows: never use of any cigar product (ie, traditional or premium, filtered, or cigarillo), ever but not current use of any cigar product, and current use of any cigar product (use every day, some days, or rarely).

c Defined as those who reported B&M as their regular or last brand for any cigar product (ie, traditional or premium, filtered, or cigarillo); all B&M smokers were current smokers.

d Missing for 16 participants (0.6%). Missing data were handled using listwise deletion.

e Missing for 17 participants (0.7%).

f Other non-Hispanic race and ethnicity included American Indian or Alaska Native, Asian, Native Hawaiian or Pacific Islander, or other not specified here.

## Data Availability

See the Supplement.
