# Supplementary material for: Association of Flavor Perception With Blue vs Purple Cigar Packaging Among US Adults
Source: JAMA Netw Open. Author manuscript; Available in PMC 2023 Mar 10. (PMC9999707; doi:10.1001/jamanetworkopen.2022.54003)
Supplement: data sharing statement [file NIHMS1872154-supplement-data_sharing_statement.pdf]

Data Sharing Statement

Delnevo. Association of Flavor Perception With Blue vs Purple Cigar Packaging Among US Adults. *JAMA Netw Open*. Published February 06, 2023.
doi:10.1001/jamanetworkopen.2022.54003

Data
Data available: No

Additional Information
Explanation for why data not available: Our informed consent did not specify secondary use of the data.
